# Complexities of nitrogen isotope biogeochemistry in plant-soil systems: implications for the study of ancient agricultural and animal management practices

**DOI:** 10.3389/fpls.2014.00288

**Published:** 2014-06-23

**Authors:** Paul Szpak

**Affiliations:** Department of Anthropology, University of British ColumbiaVancouver, BC, Canada

**Keywords:** stable isotopes, nitrogen, archaeology, agriculture, animal management

## Abstract

Nitrogen isotopic studies have the potential to shed light on the structure of ancient ecosystems, agropastoral regimes, and human-environment interactions. Until relatively recently, however, little attention was paid to the complexities of nitrogen transformations in ancient plant-soil systems and their potential impact on plant and animal tissue nitrogen isotopic compositions. This paper discusses the importance of understanding nitrogen dynamics in ancient contexts, and highlights several key areas of archaeology where a more detailed understanding of these processes may enable us to answer some fundamental questions. This paper explores two larger themes that are prominent in archaeological studies using stable nitrogen isotope analysis: (1) agricultural practices (use of animal fertilizers, burning of vegetation or shifting cultivation, and tillage) and (2) animal domestication and husbandry (grazing intensity/stocking rate and the foddering of domestic animals with cultigens). The paucity of plant material in ancient deposits necessitates that these issues are addressed primarily through the isotopic analysis of skeletal material rather than the plants themselves, but the interpretation of these data hinges on a thorough understanding of the underlying biogeochemical processes in plant-soil systems. Building on studies conducted in modern ecosystems and under controlled conditions, these processes are reviewed, and their relevance discussed for ancient contexts.

## Introduction

This paper addresses the complexities of nitrogen (N) isotopic fractionations in plant-soil systems, drawing from modern field and experimental studies to address several key areas of archaeological investigation related to prehistoric agriculture and animal husbandry. While N isotopic compositions vary in a relatively predictable and consistent manner between animal species according to trophic level (Caut et al., 2009; Szpak et al., 2012c), a diverse array of biogeochemical processes exist that influence the natural abundance of ^15^N in plant-soil systems. These processes are instrumental in structuring isotopic variation in animal tissues at various spatial and temporal scales, and it is thus imperative that isotopic studies of ancient human and animal tissues adequately consider these aspects of N isotopic biogeochemistry.

**Figure 1:**
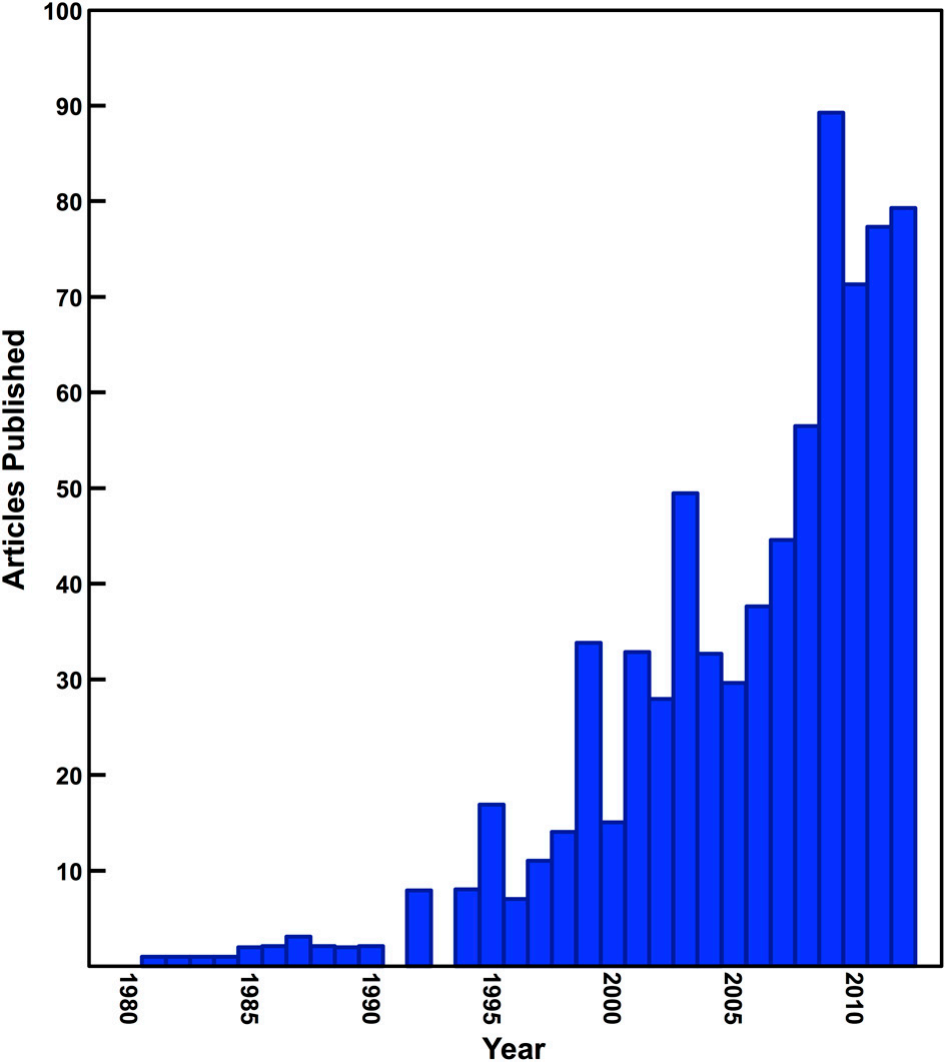
**Number of papers published in archaeology and anthropology journals utilizing isotope ratio mass spectrometry**. Data presented in this figure were generated using a simple keyword search of archaeology and anthropology journals indexed by Scopus.

Since the early 1980s, stable isotope analysis has become an extremely effective and prevalent tool for the reconstruction of the diet and ecology of human and animal species in archaeological and paleontological contexts. Stable isotope analysis is now widely used in areas of study such as foraging ecology of extinct species, large-scale shifts in ecosystems due to natural and anthropogenic processes, issues surrounding animal domestication and management, weaning behavior in past populations, agricultural practices, and the diet of prehistoric human populations in the most general sense (Schwarcz et al., 2010; Clementz, 2012). The number of archaeological studies that have used stable isotope analysis has been steadily increasing (Figure 1), but all too often the interpretations of these data rely *only* on the fundamental isotopic relationships established early on such as the differences between C_3_ and C_4_ plants or marine and terrestrial foods (DeNiro and Epstein, 1978, 1981; Schoeninger and DeNiro, 1984).

Although the isotopic data that have been and continue to be generated are overwhelmingly derived from animal tissues, the basis for the interpretation of these data are the biogeochemical processes that influence isotopic fractionations at the base of the food web. Considerable progress has been made in assessing the complexities of isotopic variation in plant-soil systems, but these processes are still only beginning to be understood in a more comprehensive manner through extensive field and laboratory studies. While several studies have been initiated by archaeologists or anthropologists (Commisso and Nelson, 2006; Bogaard et al., 2007; Fraser et al., 2011; Szpak et al., 2012a,b, 2014), most have been conducted within the context of ecology, agricultural science, food chemistry, and geochemistry. This paper synthesizes this literature, highlighting several areas of research that have direct relevance to the study of prehistoric human subsistence economies as they are assessed via stable isotope analysis. The larger goal of this work is to underscore the need for archaeologists to consider N isotopic variation in a more comprehensive manner, paying particular attention to natural and anthropogenic processes that may impact plant and soil δ^15^N values. In a more general sense, I hope that the discussion of potentially productive areas of future research that focus on better understanding N isotope dynamics can serve as a call for archaeologists and anthropologists to prioritize such work in their own research programs.

## Natural variation in plant/soil δ15N

The purpose of this paper is not to provide a detailed overview of the various processes influencing the N isotopic composition of plants and soils; for these purposes the reader is referred to many of the comprehensive reviews and syntheses that have already been published on this topic (Nadelhoffer and Fry, 1994; Handley and Scrimgeour, 1997; Högberg, 1997; Hobbie and Ouimette, 2009; Hobbie and Högberg, 2012). Nevertheless, a very brief synopsis of the major biogeochemical processes that drive variation in plant N isotopic compositions is provided below, with more detailed discussion of these processes throughout the paper where relevant.

Many factors influence plant N isotopic compositions at multiple scales. At the level of the plant, the N isotopic composition will be determined by: the type of N obtained (e.g., NO^−^_3_, NH^+^_4_, N_2_), the manner in which this N was obtained (i.e., through direct uptake of soil N or through uptake mediated by symbiotic microbes), where the N is assimilated (in the root or in the shoot), and the plant part (e.g., leaves, stem, fruit) to which the N is allocated. Beyond the level of the individual plant, foliar δ^15^N values vary across a number of spatial scales. The most significant patterns that have emerged are relationships between foliar δ^15^N and (1) plant functional type or mycorrhizal associations (Craine et al., 2009b; Hobbie and Högberg, 2012), (2) climate (Austin and Vitousek, 1998; Handley et al., 1999; Amundson et al., 2003; Murphy and Bowman, 2006), and (3) nutrient status (Stock et al., 1995; Fogel et al., 2008)—note that some of these may be strongly correlated with one another (e.g., nutrient status and mycorrhizal associations). Many studies have observed negative relationships between mean annual precipitation and foliar δ^15^N, with plants growing at arid sites being characterized by higher δ^15^N values than those growing at wetter sites (Austin and Vitousek, 1998; Handley et al., 1999; Amundson et al., 2003). Additionally, foliar δ^15^N values have been positively correlated with local temperature, such that warmer ecosystems are characterized by higher δ^15^N values than colder ecosystems (Martinelli et al., 1999; Amundson et al., 2003; Pardo et al., 2006), although this relationship deteriorates at the lowest temperatures (mean annual temperature ≤0.5°C; Craine et al., 2009b). The cause for this relationship is believed to be that hot and arid ecosystems tend to be more prone to N loss whereas colder and wetter ecosystems tend to conserve and recycle N (Handley et al., 1999). Because biogeochemical processes associated with N loss (e.g., NH_3_ volatilization and denitrification) are associated with large fractionations, enriching the remaining soil N in ^15^N, these processes drive the overall ecosystem δ^15^N values upwards. Foliar δ^15^N values have thus been used as a means of generalizing various aspects of N cycling at regional, continental, or even global scales (Amundson et al., 2003; McLauchlan et al., 2007).

**Figure 2:**
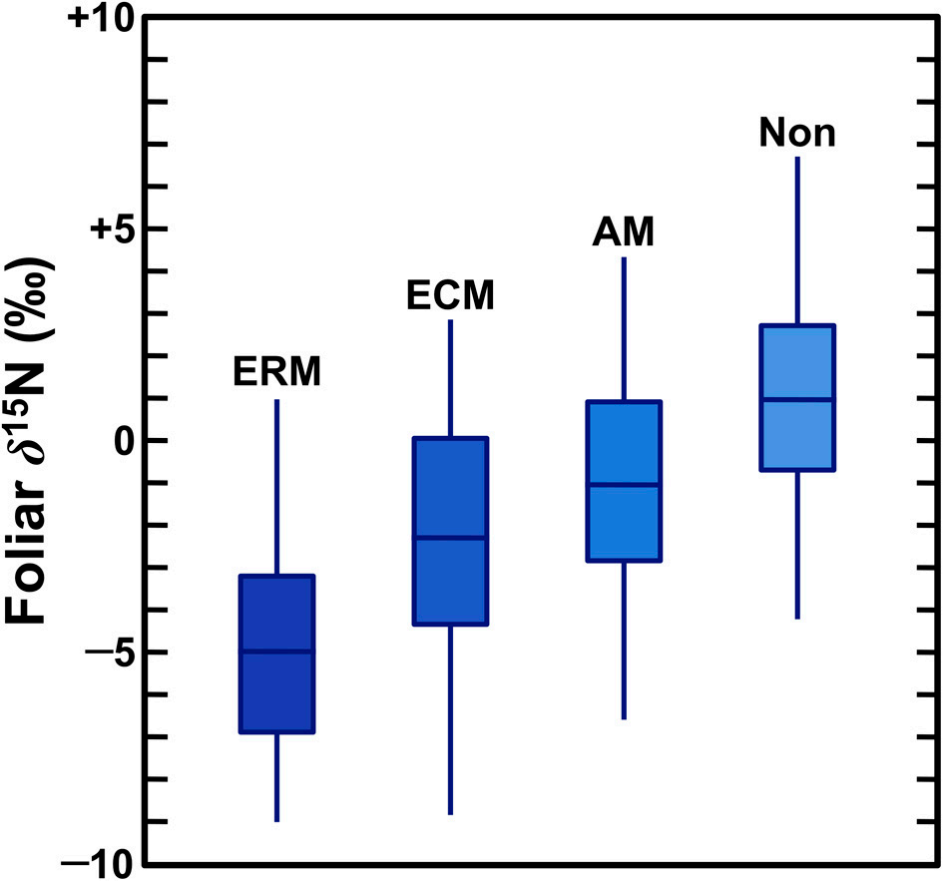
**Foliar N isotopic compositions of plants according to mycorrhizal associations (Craine et al., 2009b)**. Boxes represent interquantile (25–75%) ranges, bars dividing boxes represent means, vertical lines represent 95% of the data. As described in Craine et al. (2009b) data were normalized to a common temperature, precipitation, and foliar [N]. ERM, ericoid; ECM, ectomycorrhizal; AM, arbuscular; Non, non-mycorrhizal.

When plants acquire N through symbiotic relationships with mycorrhiza, this tends to decrease plant δ^15^N values due to the retention of isotopically heavy N by the fungi (Figure 2), although these effects vary according to the mycorrhizal type as well as local environmental conditions. Mycorrhizae are mutualistic fungi that partner with plant roots, providing plants with N (as well as other nutrients, most notably P) in exchange for photosynthates. Mycorrhizal associations are extremely important for plant communities throughout the world and most plants are dependent on mycorrhizal fungi for some portion of their N (Brundrett, 2009). The three types of mycorrhizae that are significant for this discussion are outlined below (summarized from Read, 1991; Finlay, 2008; Craine et al., 2009b; Hobbie and Agerer, 2010; Hobbie and Högberg, 2012):

1. Arbuscular mycorrhizae (AM) are the most common type and form relationships with the widest range of plant species. They are most common in areas with high rates of N cycling and lack the ability to decompose organic matter. AM plants tend to have the highest δ^15^N values of the three types, but usually have comparable or lower δ^15^N values relative to non-mycorrhizal plants.
2. Ectomycorrhizae (ECM) are common partners of long-lived perennials or trees in boreal, temperate, and to a lesser extent tropical environments. There is substantial variation in the ability of ECM to decompose organic matter. ECM with morphologies that differ according to nutrient acquisition strategy (exploration type) tend to have distinct N isotopic compositions, but overall, ECM plants have intermediate N isotopic compositions of the three mycorrhizal types.
3. Ericoid mycorrhizae (ERM) are not widely distributed, being largely limited to systems with low availability of mineralized N, low rates of N cycling, and high amounts of organic N in the soil. ERM have strong proteolytic capabilities and ERM plants tend to have the lowest δ^15^N values of the three mycorrhizal plant types.

A growing body of evidence has demonstrated that mycorrhizal fungi are enriched in ^15^N relative to their plant partners because of the transfer of ^15^N-depleted compounds to the plants (Hobbie and Högberg, 2012). A recent global survey by Craine et al. (2009b) found mycorrhizal associations significantly influence foliar N isotopic compositions, with mycorrhizal plant δ^15^N being lower than non-mycorrhizal plant δ^15^N by 2‰ for AM, 3.2‰ for ECM, and 5.9‰ for ERM. Thus, the relative importance of mycorrhizal associations plays a strong role in structuring spatial and temporal variation in plant δ^15^N values.

**Figure 3:**
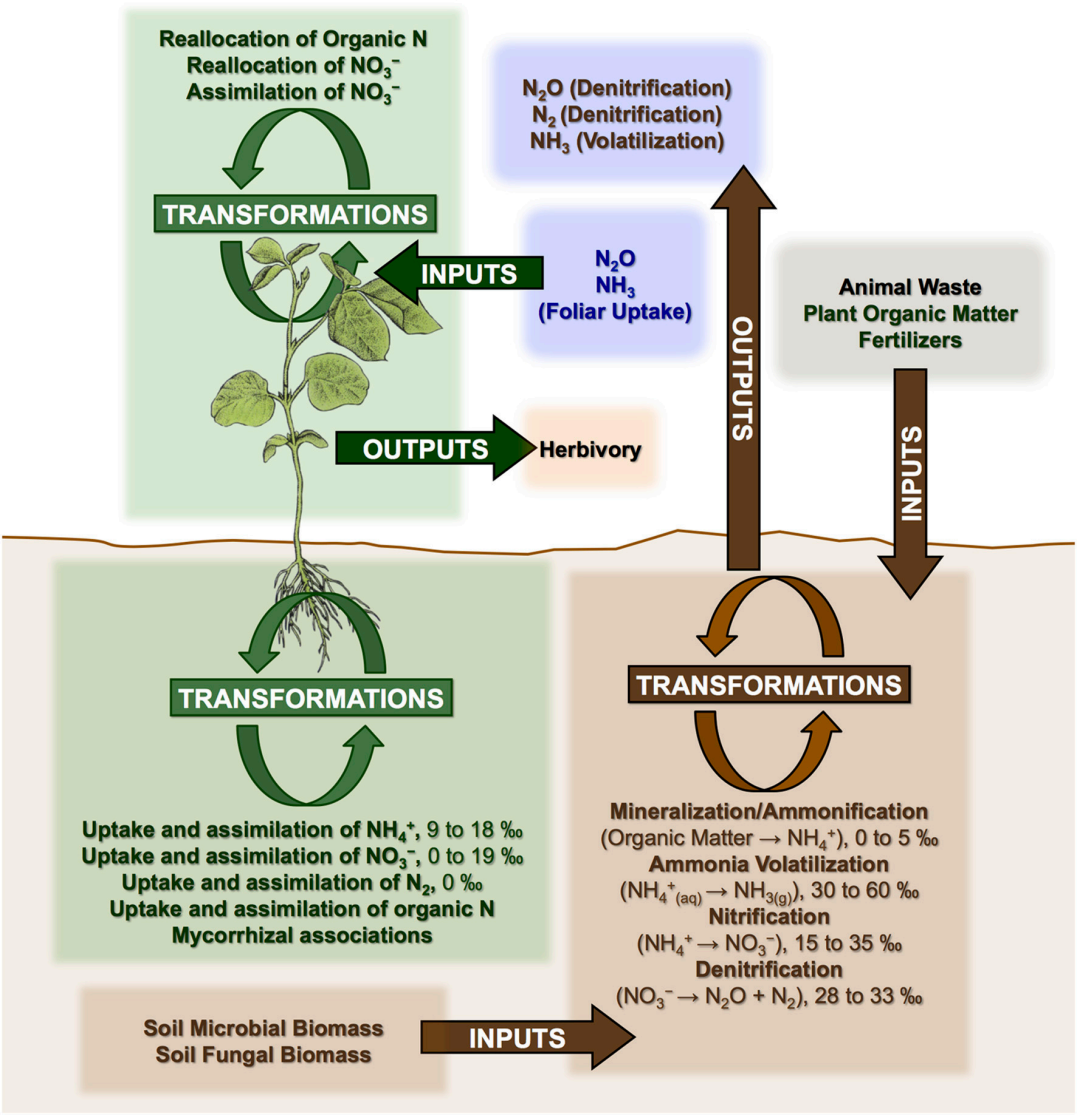
**Generalized model of N cycling in plant-soil systems showing processes that may affect plant N isotopic compositions**. Mineralization refers to the conversion of organic N into ammonium (NH^+^_4_). Nitrification refers to the conversion of ammonium into nitrate (NO^−^_3_). Denitrification is the reduction of nitrate into atmospheric nitrogen (N_2_). Fractionations (Δ^15^N) are shown for some processes (Robinson, 2001), while others with more complex and variable effects are discussed throughout the text (e.g., mycorrhizal associations, addition of animal fertilizers).

Nitrogen cycle openness and mycorrhizal associations appear to be the dominant factors controlling N isotopic variation in plants at various spatial scales (Craine et al., 2009b; Hobbie and Högberg, 2012). A generalized model of N cycling in plant-soil systems is presented in Figure 3. Three categories of processes are outlined: inputs, outputs, and transformations, all of which have the potential to influence soil and plant N isotopic compositions.

## Agricultural practices

The origins and development of agriculture have been and continue to be a topic of major theoretical and practical interest in archaeology. Because the transition to agriculture is typically coincident with a change in diet, stable isotope analysis has been utilized extensively to address the development or adoption of agriculture in various parts of the world. In those regions where the transition to agriculture involved the cultivation of a C_4_ plant (such as maize in the Americas or millet in northern China) in a predominantly C_3_ plant-dominated area, carbon isotope compositions of human or animal tissues have been of primary importance. In other areas where the suite of agricultural products were C_3_ plants (Europe and the Near East) more importance has been place on N isotopic compositions of human remains with a reduction in the importance of some food sources with relatively high δ^15^N values (e.g., marine foods, freshwater fish, animal protein in general) with the adoption of agriculture (Bocherens et al., 2007; Borić and Price, 2013). Implicit in these interpretations is an assumption that the N isotopic composition of plants, and cultivated plants in particular, is relatively static and should be approximately 3–4‰ lower than contemporaneous herbivorous animals considering typical trophic level enrichments of ^15^N (Caut et al., 2009; Szpak et al., 2012c). Recent experimental work conducted with animal fertilizers has demonstrated that this assumption is an oversimplification and somewhat problematic in agricultural societies. This section will review three processes that were likely important in prehistoric agriculture (use of animal fertilizers, burning of vegetation or shifting cultivation, and tillage) and that have the potential to alter plant and soil N isotopic compositions.

### Animal fertilizers

The maintenance of soil fertility has been, and continues to be, a matter of utmost importance to agricultural societies. Fertilizers derived from animal excreta have long been significant sources of nutrients (including N) for cultivated plants at a global scale, likely for millennia (Jones, 2012). In prehistoric contexts, the detection of the use of animal-derived fertilizers is not straightforward and is often ambiguous, although several lines of evidence have been utilized to detect the presence of animal dung in archaeological deposits (see chapters in Jones, 2012). Nitrogen is the most important nutrient added to the soil via animal manure, and the N isotopic composition of animal manure is often higher than that of endogenous soil N. There is, therefore, potential for plant N isotopic compositions to be significantly altered by fertilization. This has important implications for the interpretation of isotopic data derived from human and animal remains, as well as for the potential detection of fertilization practices in prehistoric contexts.

**Figure 4:**
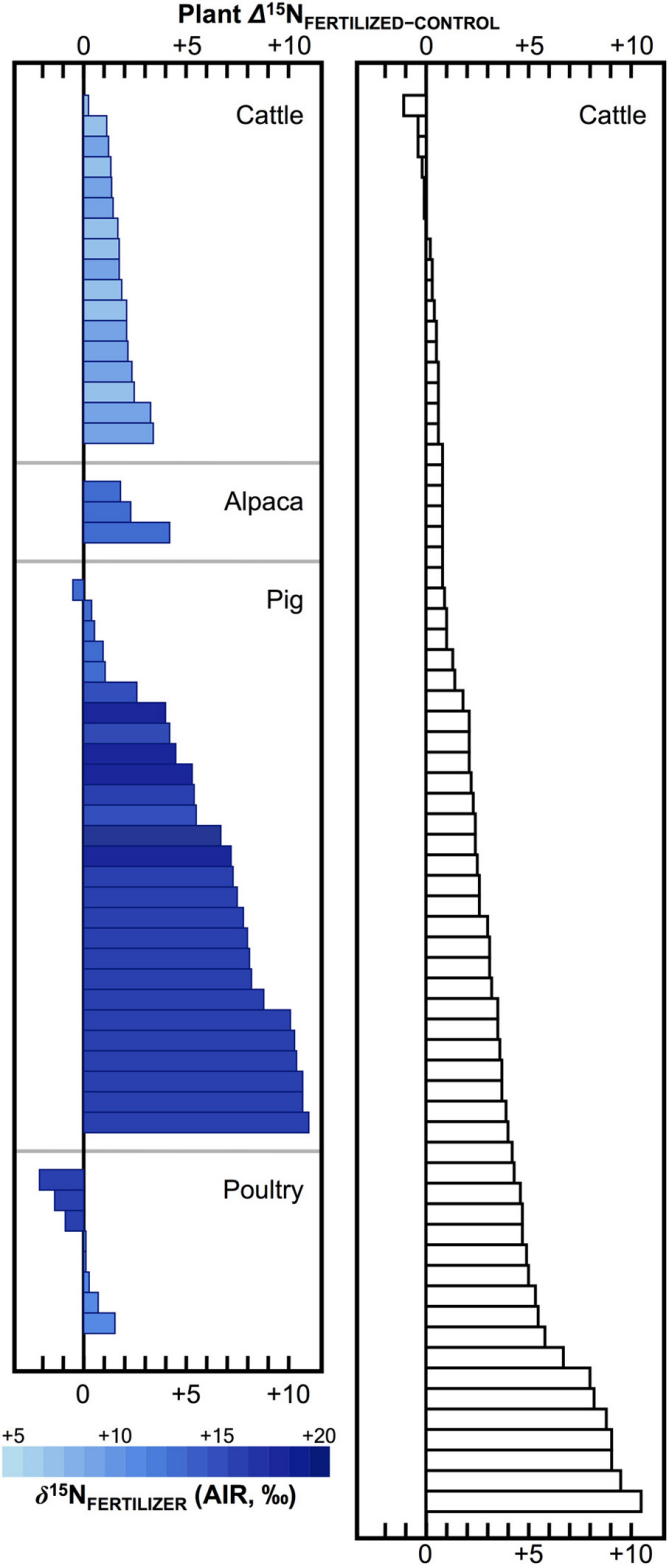
**Effects of animal fertilizers on plant δ^15^N values**. Bars represent differences between control plants (no fertilizer applied) and treatments receiving animal fertilizers. Bars are shaded according to δ^15^N of the fertilizer applied, with darker colors representing higher δ^15^N values; open bars indicate fertilizer δ^15^N value was not determined (right panel). Data are compiled from published literature (Choi et al., 2002; Watzka et al., 2006; Yun et al., 2006; Lim et al., 2007; Yun and Ro, 2009; Fraser et al., 2011; Szpak et al., 2012b; Yuan et al., 2012; Zhou et al., 2013). Studies contrasting chemically and organically fertilized plants without unfertilized controls were not included. For readability, results from plants fertilized with seabird guano (differences between fertilized and control plant δ^15^N +20 to +40‰) were excluded (Szpak et al., 2012a,b, 2014).

**Figure 5:**
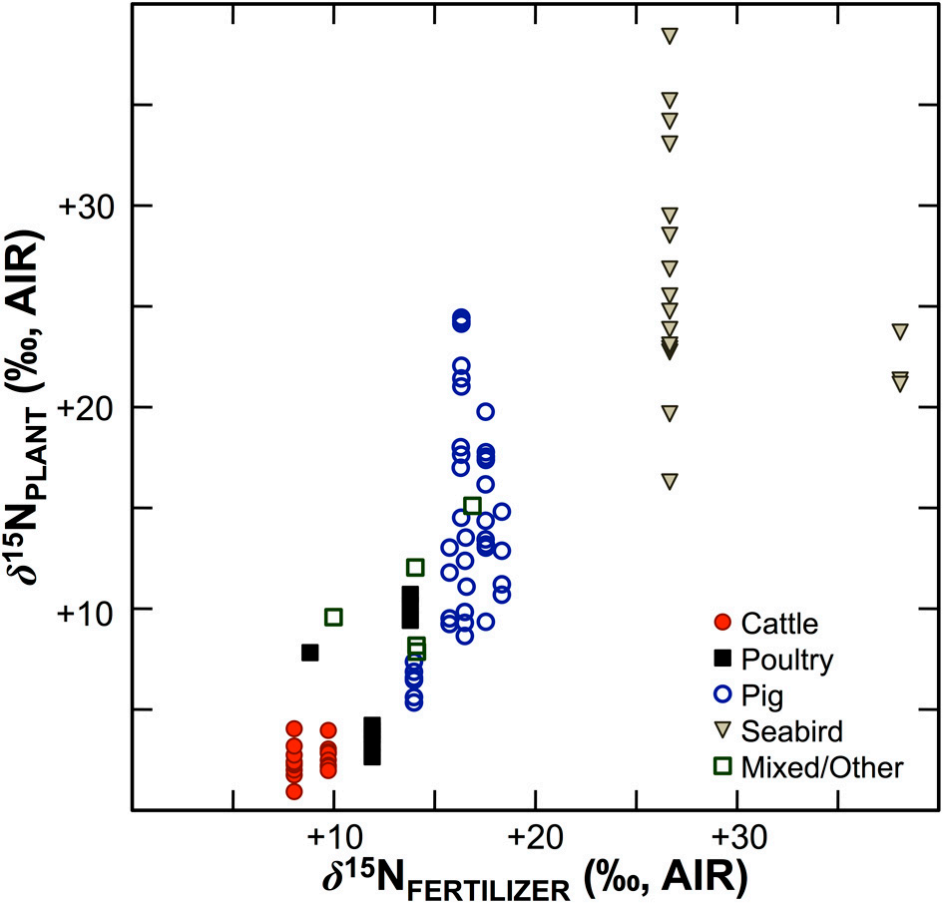
**Bivariate plot of fertilizer δ^15^N vs. plant δ^15^N**. Generally, plant δ^15^N increases with fertilizer δ^15^N, although there is considerable variation, which can be ascribed to the relative availability of N compounds in the fertilizer, the rate and duration of application, and interspecific differences in plant N acquisition strategies. Data are compiled from experimental studies (Choi et al., 2002, 2003; Watzka et al., 2006; Yun et al., 2006; Lim et al., 2007; Nakano and Uehara, 2007; Yun and Ro, 2009; Rapisarda et al., 2010; Szpak et al., 2012a,b, 2014; Yuan et al., 2012; Zhou et al., 2013).

Numerous studies in the fields of soil and plant sciences (Choi et al., 2002, 2003, 2006; Watzka et al., 2006; Yun et al., 2006; Lim et al., 2007; Yun and Ro, 2009; Kriszan et al., 2014), agricultural and food chemistry (Bateman et al., 2005; Nakano and Uehara, 2007; Del Amor et al., 2008; Rapisarda et al., 2010; Yun et al., 2011; Yuan et al., 2012; Zhou et al., 2013), and archaeology (Bogaard et al., 2007; Fraser et al., 2011; Szpak et al., 2012b) have demonstrated higher δ^15^N values for plants fertilized with animal manures relative to unfertilized plants, or plants treated with nitrogenous chemical fertilizers (Figure 4). The extent to which animal fertilizer application affects plant δ^15^N values is highly variable and depends on the type of fertilizer applied, the amount applied, and the duration of application. For the animal fertilizers that have been studied, a pattern of ^15^N enrichment in plants can be summarized as follows: poultry ≤ cattle < pig < seabird (Figure 5). In general, as the δ^15^N value of the manure increases, so too does the δ^15^N value of the fertilized plant. It is important to keep in mind, however, that the *input* of a new N source that occurs with fertilization is not the only factor that affects the δ^15^N values of soils and plants—the variation observed in plant δ^15^N for any particular fertilizer δ^15^N clearly demonstrates the importance of other factors (Figure 5). The extent to which a particular fertilizer will be expected to influence the N isotopic composition of a plant will also be strongly dependent on how the presence of that fertilizer impacts transformations and losses (outputs) of N, since these processes discriminate against ^15^N, in some cases very strongly (Figure 3). For instance, while the δ^15^N values of pig manure is typically higher than the δ^15^N values of cattle manure (Bateman and Kelly, 2007), the availability of mineralized N from pig manure is also much higher than for cattle manure—20 to 40% N available from cattle manure after 1 year compared to 75–90% for pig manure (Eghball et al., 2002). While the mineralization of organic N is not associated with any appreciable fractionation (Robinson, 2001), the availability of the fertilizer-derived N will be strongly affected by mineralization rates.

The bulk N isotopic composition of mammalian urine tends to be depleted in ^15^N relative to the diet by about −2.5‰, while feces is typically enriched in ^15^N relative to the diet by +2.0‰ (Table 1). The δ^15^N values of animal manures typically are not consistent with the diet-feces or diet-urine ^15^N fractionations that have been observed for many species, and instead manures tend to be characterized by much higher δ^15^N values than would be expected. This is because of several important chemical processes that act on various N species in these manures at various stages between animal excretion, collection, composting (if applicable), storage, and decomposition after application. These processes include: ammonification (mineralization), immobilization, nitrification, dentrification, NH_3_ volatilization, and leaching (Petersen et al., 1998). Nitrogen losses from NH_3_ volatilization and denitrification tend to be particularly large during manure storage, with losses of 10–40% N being common (Kirchmann, 1985). Accordingly, there tends to be a considerable increase in manure δ^15^N values during composting and storage (Choi et al., 2007; Kim et al., 2008).

**Table 1:** **Summary of studies presenting data for the differences in N isotopic compositions between the diet and urine and/or diet and feces (Δ^15^N) of different mammalian species**.

**Species**	**Urine Δ^15^N (‰, AIR)**	**Feces Δ^15^N (‰, AIR)**	**References**
Deer mouse		+2.1	Hwang et al., 2007
Red-backed vole		+2.2	Hwang et al., 2007
Long-tailed vole		+2.2	Hwang et al., 2007
Meadow vole		+2.5	Hwang et al., 2007
Yellow-pine chipmunk		+1.4	Hwang et al., 2007
Western jumping mouse		+2.2	Hwang et al., 2007
Greater mouse-eared bat		+1.5	Salvarina et al., 2013
Greater horseshoe bat		+1.5	Salvarina et al., 2013
Llama	−0.3	+2.9	Sponheimer et al., 2003
Llama	−2.1	+3.0	Sponheimer et al., 2003
Horse	−0.1	+2.6	Sponheimer et al., 2003
Horse	−2.0	+3.3	Sponheimer et al., 2003
Sheep	−0.1	+3.1	Sutoh et al., 1993
Sheep		+3.0	Wittmer et al., 2011
Caribou	+0.2		Gustine et al., 2011
Musk ox	−2.9		Gustine et al., 2010
Goat		+3.6	Sutoh et al., 1987
Goat		+1.7	Codron et al., 2012
Cow	−4.0		Knobbe et al., 2006
Cow		+2.0	Steele and Daniel, 1978
Cow		+2.0	Steele and Daniel, 1978
Cow		+2.0	Steele and Daniel, 1978
Cow		+1.7	Steele and Daniel, 1978
Cow	−4.0	+0.6	Sutoh et al., 1987
Cow	−5.3	+0.4	Sutoh et al., 1987
Cow	−5.1	+1.4	Sutoh et al., 1987
Pig	−2.6	+1.2	Sutoh et al., 1987
White-tailed deer		+2.1	Nájera-Hillman and Mandujano, 2013
Average	−2.4 ± 2.0	+2.1 ± 0.8	

While the potential effects of animal fertilizers on δ^15^N values in plant-soil systems have long been known (Riga et al., 1971; Kreitler and Jones, 1975), it has only been since the recent work of Bogaard et al. (2007) on the N isotopic compositions of plants from long-term agricultural stations that archaeologists have begun to more seriously consider the potential impact of fertilizers on plant, and in turn, human tissue δ^15^N values. Two key issues in archaeology have emerged out of this recent reconsideration of the effects of animal fertilizers on plant δ^15^N values, one a complication and one an opportunity. The complication comes with the interpretation of human δ^15^N values from archaeological contexts. Essentially, because of the reasonably consistent trophic level enrichment in ^15^N that occurs between herbivores and plants (Caut et al., 2009), all things being equal, herbivorous animal tissues should have δ^15^N values 3–4‰ higher than coeval plants. Working backwards from this general relationship, direct measurements of contemporaneous herbivore bone collagen δ^15^N values are used as a reference point for crop δ^15^N values. Considering a scenario where animal manure is used to fertilize plants and the δ^15^N values of those plants are 2–6‰ higher than would be expected without the addition of the manure there is a convergence of plant and animal δ^15^N values (provided the animals are not consuming significant quantities of fertilized crops) such that the relative contributions of plant and animal protein to the diet become less clear. Bulk collagen δ^15^N values of human bone collagen are thus insufficient in and of themselves to differentiate between terrestrial herbivore and fertilized plant consumption. Accordingly, additional isotopic markers are required. The isotopic analysis of individual amino acids isolated from bone collagen is particularly promising in this regard as there is evidence that the consumption of plant vs. animal protein can be resolved via the carbon isotopic composition of individual amino acids (Petzke et al., 2005), which should not be influenced by fertilization (Szpak et al., 2012a).

When considering N isotopic data derived from human tissues such as bulk bone collagen, direct evidence for the manuring of crops will be ambiguous. There is opportunity to more directly approach questions of crop management and manuring practices through the analysis of plant material preserved at archaeological sites. DeNiro and Hastorf (1985) demonstrated the potential of analyzing the N isotopic composition of charred plant remains for paleodietary baselines. More recently, a number of studies have utilized similar methods to investigate the manuring of ancient cereals, as well as various aspects of crop management that may affect plant δ^15^N values, at European and Near Eastern sites (Lightfoot and Stevens, 2012; Bogaard et al., 2013; Vaiglova et al., 2014; Kanstrup et al., in press). Interpreted within the context of data from long-term experimental agricultural stations, these data have indicated variable levels of manuring over thousands of years, although it is not possible to draw a direct causal link between the application of animal manure and elevated archaeobotanical δ^15^N values given the highly variable nature of plant N isotopic compositions, even at relatively small spatial scales. Experimental charring has demonstrated that this process does not substantially alter grain δ^15^N values (Kanstrup et al., 2012; Fraser et al., 2013; Styring et al., 2013), but it is less clear to what extent post-depositional processes may alter these values, if at all. Comparatively, a range of techniques and measures are available for assessing the “intactness” of collagen extracted from bone (C:N ratio, minimum %C and %N, collagen yield), which can be used to discard data unlikely to be representative of endogenous isotopic compositions. No such measures currently exist for charred plant remains and this no doubt has to do with the non-specific nature of bulk plant samples (relative to purified protein isolated from bone). Recent work by Styring et al. (2013) is an important first step in this direction, but additional studies are required to develop a more comprehensive and reliable set of quality indicators.

### Effects of burning/shifting cultivation

One of the most frequently discussed forms of land management by prehistoric human populations is swiddening (alternatively slash-and-burn or shifting cultivation). Vegetation is cleared by burning and crops are grown on the land until soil fertility sufficiently declines and this land is left fallow for a number of years and allowed to regenerate; this pattern is shifted to another location and repeated. It has been suggested that swidden agriculture was important in nearly every region of the globe where plants were cultivated prehistorically, and although its significance has been questioned in some parts of the world (Sherratt, 1980; Bogaard, 2002), swidden agriculture remains a prominent aspect of cultivation in the humid tropics today and was likely of some importance prehistorically even if not at a global scale. In addition to farmers, clearing vegetation through burning is also a behavior recorded for numerous groups of foragers to encourage the presence of particular species (Rowley-Conwy and Layton, 2011). Thus, within the context of human dietary studies, this issue requires consideration. Numerous studies have examined the potential consequences of vegetation burning on soil and plant N isotopic compositions (Herman and Rundel, 1989; Mordelet et al., 1996; Grogan et al., 2000; Cook, 2001; Saito et al., 2007; Schafer and Mack, 2010; Beghin et al., 2011; Johnson et al., 2011; Huber et al., 2013; Leduc et al., 2013), but to the best of my knowledge these effects have not been investigated directly as they are associated with swidden agriculture and are instead mostly limited to studies of forest soils that are not cultivated after burning. Nevertheless, some generalizations about N cycling in burned soils can be made.

In the process of clearing land by burning, there are alterations to the distribution and cycling of nutrients. Ash that is incorporated into the soil is typically rich in P and mineral nutrients (e.g., K, Ca, Mg), with C and N being largely lost due to volatilization (Juo and Manu, 1996). Aside from direct nutrient additions to the soil, the addition of charred organic material has been demonstrated to facilitate the retention of soil nutrients (such as those added by organic fertilizers) in both temperate (Laird et al., 2010) and tropical (Lehmann et al., 2003) environments. Although burning results in large losses of N, soil mineralized N pools (NH^+^_4_ especially) have been observed to increase significantly after burning (Grogan et al., 2000; Schafer and Mack, 2010; Huber et al., 2013). There are several possible reasons for this increase: (1) N is added directly through the contribution of burned vegetation, although this is not supported in most cases because the N content of the ash itself tends to be extremely low, (2) increased temperature in the soil enhances mineralization of organic N in the soil (Klopatek et al., 1990), and (3) the increase in soil pH caused by the addition of ash may enhance microbial activity (Grogan et al., 2000). The mechanism(s) by which additional mineralized N enters the burned area is significant because they will have variable consequences on the N isotopic composition of the soil N pools and in turn on foliar δ^15^N values. With respect to the organic material derived from the burned vegetation, the duration and intensity of the fire may have important consequences for the N isotopic composition of the resultant ash or char. For very high temperature fires, it has been suggested that there is little opportunity for fractionation because nearly all of the N is lost in gaseous form (Saito et al., 2007). Conversely, for low temperature fires resulting in charred organic material rather than ash, there is greater opportunity for fractionation and the charred material may be enriched in ^15^N relative to unburned vegetation (Saito et al., 2007; Huber et al., 2013). Therefore, where vegetation is relatively N rich and fire intensity is relatively low, the mineralized N derived from this organic matter may be enriched in ^15^N relative to the overall soil N pool.

Numerous studies have examined the consequences of vegetation burning on foliar and soil δ^15^N values with inconsistent results. Most studies have recorded either significantly higher foliar δ^15^N values in post-fire vegetation (Grogan et al., 2000; Saito et al., 2007; Beghin et al., 2011; Johnson et al., 2011; Huber et al., 2013; Leduc et al., 2013) or inconsistent changes between pre-and post-fire vegetation (Mordelet et al., 1996; Schafer and Mack, 2010; Beghin et al., 2011); comparatively few studies have found significantly lower foliar δ^15^N values following fire (Herman and Rundel, 1989; Cook, 2001). That there is not a consistent pattern in all cases is not surprising and likely reflects the diversity of processes responsible for the N available, which will vary on a case-by-case basis. Nonetheless, higher δ^15^N values in post fire vegetation initially, followed by a return to pre-fire δ^15^N values is the most common pattern recorded.

Two hypotheses have been put forth to explain the elevated foliar δ^15^N values following fire, which are not mutually exclusive. First, as discussed above, the burning of organic material may cause discrimination against ^15^N, leaving the residual organic matter relatively ^15^N enriched (Saito et al., 2007). This organic matter is mineralized in the soil to NH^+^_4_ and taken up by plants. As nitrification, which discriminates against ^15^N, proceeds, the NH^+^_4_ pool becomes more ^15^N-enriched and creates a situation where plants that use predominantly NH^+^_4_ are characterized by significantly higher δ^15^N values (Huber et al., 2013). Additionally, although both NH^+^_4_ and NO^−^_3_ are highly soluble, NH^+^_4_ has a greater capacity to adsorb to organics and minerals, and is thus less likely to be lost due to leaching (Johnson et al., 2011), which may further enriched the post-fire N pool in ^15^N. The second hypothesis was outlined by Högberg (1997) and focuses on the differential access of organic matter pools with variable depth in burned and unburned soils. In short, fire consumes the surface organic matter layer, which tends to be depleted of ^15^N relative to deeper horizons, especially in forest soils (Nadelhoffer et al., 1996; Hobbie and Ouimette, 2009). Following a fire, plants rely on mineralized N from deeper horizons, which is enriched in ^15^N relative to the litter, causing their tissues to also be enriched in ^15^N (Högberg, 1997). After vegetation reestablishes in the burned zone, foliar δ^15^N values gradually return to pre-fire levels (Leduc et al., 2013).

**Figure 6:**
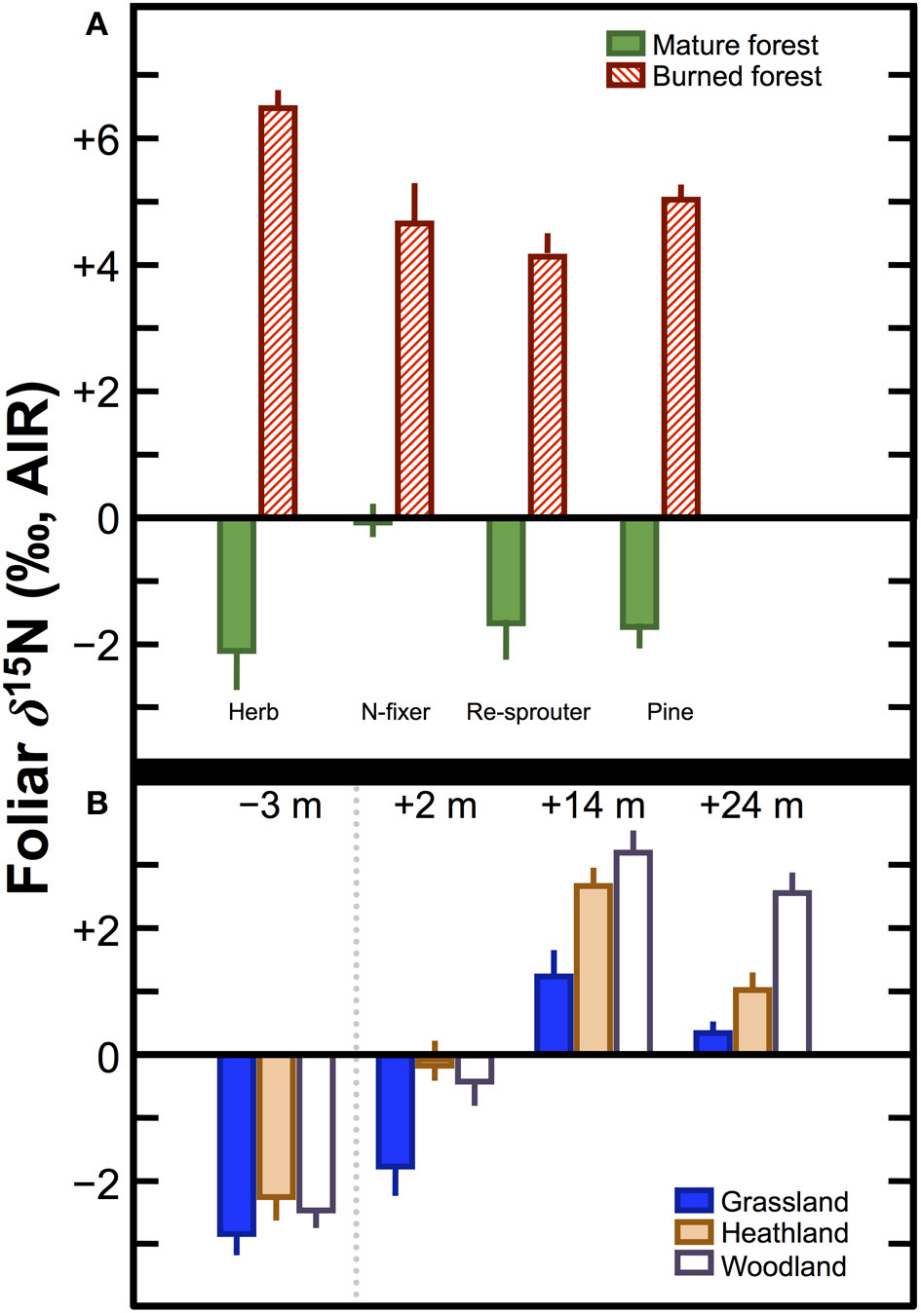
**Effects of fire on plant *δ*^15^N values**. **(A)** Comparisons between plants grown on mature and recently burned California pine forests (Grogan et al., 2000). **(B)** Change over time in three different Australian ecosystems following substantial wildfires. Values above indicate number of months prior to or after the wildfires. The broken vertical line separates the pre- and post-fire samples (Huber et al., 2013). In both **(A,B)** bars represent means ± one standard deviation.

The magnitude of the difference observed in δ^15^N between pre- and post-fire vegetation, or between vegetation on control plots and plots subjected to fire can be quite large (Figure 6). For example, in California pine forest stands, Grogan et al. (2000) recorded differences in foliar δ^15^N between burned and mature forests of +8.6‰ (herb), +4.7‰ (N-fixer), +5.8‰ (re-sprouter), and +6.0‰ (pine). Approximately 1 year after wildfires in Australia, Huber et al. (2013) found increases in foliar δ^15^N relative to pre-fire values of: +4.1‰ (grassland), +4.8‰ (heathland), +5.5‰ (woodland). These differences in foliar δ^15^N are comparable to, or in some cases greater than, those reported between unfertilized plants and those fertilized with cattle manure. Accordingly, the potential impact of burning on crop δ^15^N values within the context of shifting cultivation requires additional investigation. Although the effects of burning on foliar δ^15^N values lessen after a period of several years or decades (Leduc et al., 2013), the relatively short period during which burned plots would be utilized in shifting cultivation (typically 1–5 years) (Bogaard, 2002) creates a strong possibility that plant δ^15^N values could be significantly altered in a systematic manner.

### Tillage

The mixing of soil using plow technology was an important part of agriculture in the Old World from at least the fourth millennium B.C. (Sherratt, 1980). Even in the absence of draft animals, mechanical agitation of the soil through human tillage has likely been an important part of cultivation globally. The mechanical agitation of soil that occurs with tillage serves to disturb aggregated soil particles, increases aeration, and exposes previously buried soil surfaces (Erickson, 1982). Thus, in addition to bringing mineralized nutrients to the surface directly, tillage promotes mineralization of organic matter (Silgram and Shepherd, 1999). These practices may have significant implications for the N isotopic compositions of soils and plants because there is often strong variation in δ^15^N according to depth within a soil profile (Hobbie and Högberg, 2012). An increase in soil δ^15^N with depth has been observed in forests (Gebauer et al., 1994; Högberg et al., 1996; Koopmans et al., 1997; Emmett et al., 1998), grasslands (Steele et al., 1981; Mordelet et al., 1996; Frank and Evans, 1997), tundra (Nadelhoffer et al., 1996), and pastures (Steele et al., 1981; Ledgard et al., 1984; Piccolo et al., 1996). The magnitude of these changes appears to be consistently the greatest in forests relative to other environments (Hobbie and Ouimette, 2009). In some cases, although deeper layers are generally characterized by higher δ^15^N values than more shallow layers, a maximum δ^15^N value is reach at an intermediate depth in the soil profile (e.g., 20 cm) (Hobbie and Ouimette, 2009). Because of this depth-related variation in soil δ^15^N, it has been suggested that differences in rooting depth between plant species or functional types are responsible for some variation in wild plant δ^15^N values (Schulze et al., 1994), although these effects have not been investigated in agricultural contexts. Several mechanisms have been suggested to be responsible for the increase in soil δ^15^N values with depth:

1. Plant tissues are depleted in ^15^N relative to surface soils and the return of ^15^N-depleted plant matter to the soil surface via litterfall creates a ^15^N-depleted surface layer (Högberg, 1997).
2. Soil δ^15^N increases with the age of organic matter and the extent of decomposition. As organic matter undergoes mineralization, the ammonium that is produced is relatively depleted in ^15^N and the residual organic matter becomes increasingly ^15^N-enriched. Organic matter decomposes, reduces in size, and travels down the soil profile, creating the characteristic increase in δ^15^N with depth (Tiessen et al., 1984; Nadelhoffer and Fry, 1994).
3. Through mycorrhizal transfer, plants acquire N that is relatively depleted in ^15^N and once incorporated into the litter layer, this decomposing plant material creates a ^15^N-depleted surface. Nitrogen derived from the tissues of the mycorrhizal fungi, which are enriched in ^15^N relative to their plant partners, accumulates at greater depths, leading to ^15^N-enriched soil N relative to the surface layers (Högberg et al., 1996; Hobbie et al., 1999; Hobbie and Ouimette, 2009; Hobbie and Högberg, 2012).

**Figure 7:**
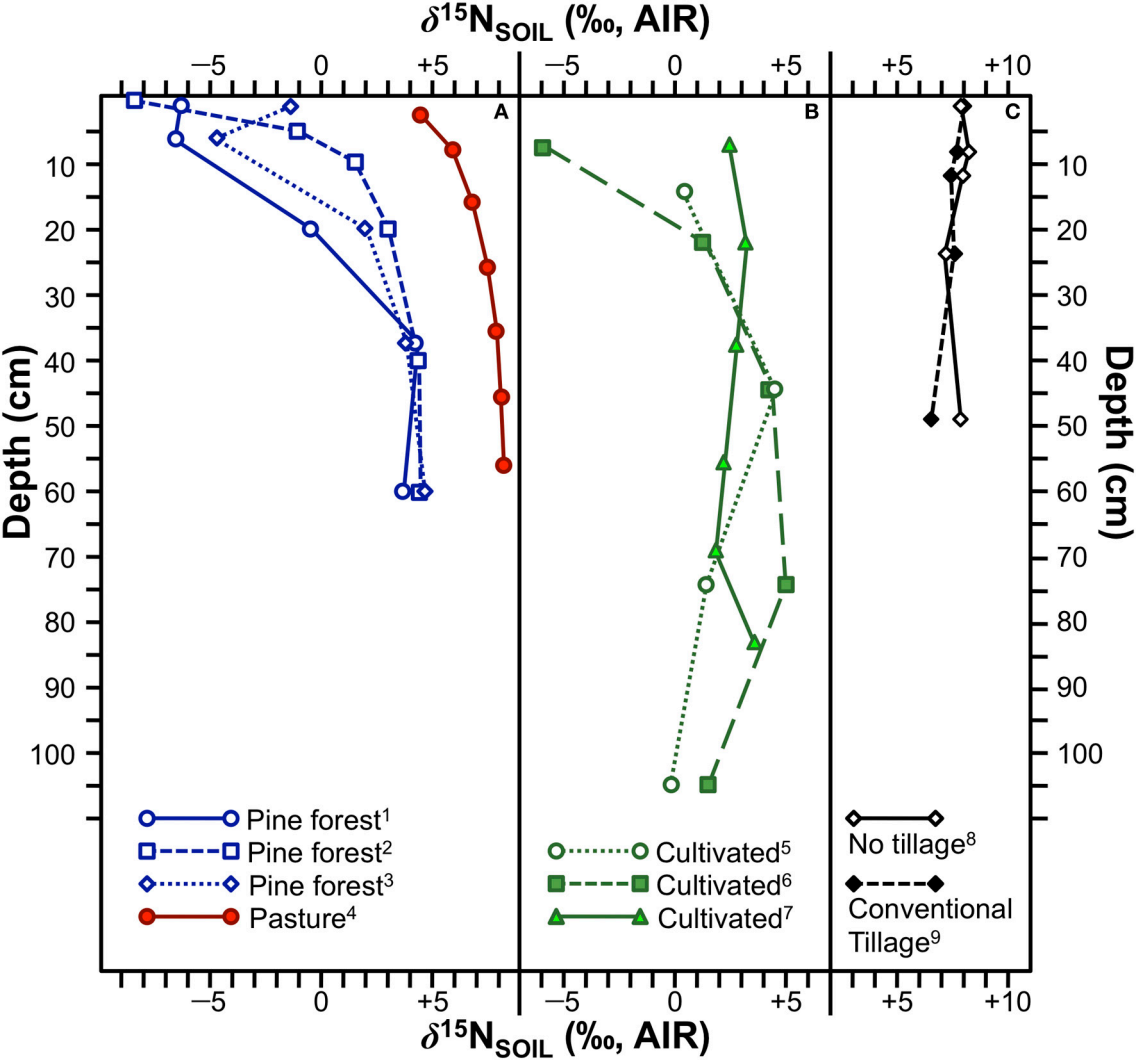
***δ*^15^N variation with depth in soil profiles under various conditions**. **(A)** Soil profiles for three pine forests and one pasture. The much larger and abrupt shift in δ^15^N with depth is typical of N-limited forests. (1) pine forest, Netherlands (data from Koopmans et al., 1997), (2) pine forest, USA (redrawn from Hobbie and Ouimette, 2009), (3) pine forest, Netherlands (data from Koopmans et al., 1997), (4) native pasture, southeastern Australia (redrawn from Ledgard et al., 1984). **(B)** Soil profiles for three cultivated California soils. (5) Hanford sandy loam, (6) Yolo fine sandy loam, (7) Vernalis silt loam (data from Broadbent et al., 1980). **(C)** Comparison of soil profiles from experimental fields in Saskatchewan with no tillage (8) and conventional tillage (9) showing no significant differences in δ^15^N between the two fields (data from Selles et al., 1984).

The redistribution of N throughout the soil profile associated with tillage has the potential to disturb the depth-related variation in soil δ^15^N. Specifically, the exposure of deeper layers that are likely enriched in ^15^N to the surface might cause plants growing in tilled fields to be characterized by higher δ^15^N values than those growing in no-till fields (see Emmett et al., 1998), although there are several issues with this notion. For the first of the three hypotheses discussed above (^15^N-depleted litterfall creates a ^15^N-depleted soil surface horizon), the incorporation of plant matter into a discrete layer of surface litter occurs in only limited circumstances as much of this above-ground organic matter is removed during harvest, or is redistributed through the soil profile during tilling. Thus, the abrupt shift in δ^15^N in the uppermost portions of the soil profile associated with forests (Figure 7A) is unlikely to occur in most cultivated fields (Figure 7B), and this is generally consistent with field measurements (Shearer et al., 1978; Karamanos et al., 1981; Selles et al., 1984).

Mycorrhizal associations (specifically arbuscular) are critical components of sustainable and organic farming systems (Gosling et al., 2006). The majority of agricultural plants with the exceptions of the Brassicaeceae (e.g., broccoli, cabbage, radish) and Chenopodiaceae (e.g., spinach, quinoa) form mycorrhizal associations (Newman and Reddell, 1987). That agricultural fields tend to be overwhelmingly characterized by arbuscular mycorrhizal communities is significant. The majority of temperate forests from which significant changes in soil δ^15^N with depth have been recorded are dominated by ectomycorrhizae, which tend to be more ^15^N enriched (and hence symbiotic plants are more ^15^N depleted) relative to AM (Craine et al., 2009b; Hobbie and Högberg, 2012). Communities dominated by ectomycorrhizae tend to be characterized by larger changes in soil δ^15^N with depth relative to arbuscular-dominated communities (Hobbie and Ouimette, 2009). Additionally, the increased N or P input through fertilization that characterizes many agricultural fields is likely to inhibit mycorrhizal colonization (Jensen and Jakobsen, 1980; Kahiluoto et al., 2001), although this may not apply equally well to the relatively low-input farming of the past. Further, the act of tilling the soil can decrease the prevalence of mycorrhizal root colonization and the contribution of mycorrhizae to overall fungal biomass in agricultural fields (Kabir et al., 1997; Mozafar et al., 2000; Van Groenigen et al., 2010). Thus, from a mycorrhizal perspective the variation in soil δ^15^N with depth should not be as pronounced in agricultural fields relative to forests, both for tilled and no-till soils.

In agricultural fields the increase in soil δ^15^N with depth is unlikely to be as strong as in forests and systematic depth-related variation in δ^15^N may be completely absent, reducing the likelihood that tillage would significantly increase plant δ^15^N values by bringing ^15^N-enriched compounds to the surface. Selles et al. (1984) tested this premise directly and found no significant change in soil δ^15^N at any depth due to tillage (Figure 7C). It is therefore unlikely that soil tillage is a significant contributor to variation in ancient plant N isotopic compositions.

### Areas for future research

A critical area of investigation within the context of human paleodietary studies involves understanding the range and variation of foods that may have been consumed. Most studies that have attempted to differentiate organically and chemically fertilized crops have found significantly lower δ^15^N values in the plants treated with chemical fertilizers (Bateman et al., 2005; Choi et al., 2006). Given the importance of chemical fertilizers in modern agriculture (Matson et al., 1997), this must be taken into account when data derived from modern surveys of cultivated plants are used as dietary baselines (e.g., Keegan and DeNiro, 1988; Szpak et al., 2013; Warinner et al., 2013). Many of the N isotopic compositions derived from modern plants may be of little or no value as paleodietary baselines if the fertilization method used is unknown or relies on chemical N fertilizers. Interpretations utilizing currently published data must therefore interpret these δ^15^N values cautiously.

Additional studies in controlled and field settings are essential to better understand the complexities of N cycling in ancient agricultural systems and their consequences on plant and soil N isotopic compositions. To do so, archaeologists must initiate controlled and field studies that have the greatest potential to yield results with direct relevance to archaeological contexts. In this respect, there is considerable potential for biogeochemically-oriented archaeologists to conduct collaborative research with ethnobiologists studying traditional land management systems. Moreover, the growing interest in organic and sustainable farming systems creates an additional layer of relevance to the investigation of nutrient cycling in ancient agricultural contexts. Importantly, we must not maintain a myopic focus on animal fertilization, and should instead look to a more diverse array of land management strategies and their effects on soil and plant N isotopic compositions.

To understand the nature of agricultural practices in the past, the greatest potential exists with the analysis of archaeobotanical materials as they provide a more direct (relative to animal tissues) view of N cycling. To effectively make sense of these data, however, additional work is required to resolve a wider range of factors that may influence cultigen N isotopic compositions. Additionally, where the interpretation of a particular agricultural practice (e.g., manuring) rests on the difference of a few ‰ in δ^15^N, we must be able to assess with a high degree of confidence whether the N isotopic compositions derived from archaeobotanical materials are in fact endogenous and not influenced by post-depositional alteration.

## Animal domestication and husbandry

The nature of human-animal relationships has been an area of intensive study, both in anthropology and archaeology. The manner in which these relationships develop and change over time in domestic animals is particularly significant, and isotopic analysis has figured prominently in this context in recent years. Isotopic data have been used to examine specific types of plants consumed by animals, the scale of animal herding, demography, and the trade in animal products. The majority of domestic animals from archaeological contexts that have been subjected to isotopic analysis are herbivores (e.g., cattle, sheep, goats, camelids). Therefore, unlike omnivorous species (e.g., dogs, pigs), the N isotopic compositions of herbivore tissues will not be affected by trophic level, but principally by biogeochemical processes in plant-soil systems. It is therefore important to consider how these systems may be different for animals kept under different conditions (e.g., stabling vs. free-ranging) or fed particular diets (e.g., agricultural byproducts). This section focuses on the isotopic consequences of grazing intensity/stocking rate and the foddering of domestic animals with cultigens.

### Grazing intensity/stocking rate

The presence of grazing animals has the capacity to alter nutrient cycling in plant-soil systems through several mechanisms: additions of nutrients derived from urine and feces, trampling and physical disturbances, and changes in floral community composition (Bardgett and Wardle, 2003; Singer and Schoenecker, 2003). Within the context of N isotope studies, the effects of herbivore waste on plant-soil systems have been studied in a number of natural and controlled settings. While this section concentrates on grazing intensity in domestic herbivore species, it is also applicable to wild species and has implications for paleoecological contexts.

Where animals deposit waste, the concentrated addition of mineral nutrients and organic matter to the soil has the potential to alter the N isotopic compositions of soils and plants. This is somewhat analogous to the effects of animal fertilizers on plant δ^15^N values in agricultural fields although the redistribution of N through herbivore activity is qualitatively and quantitatively different from the direct application of animal manure. Several archaeological studies have discussed the possibility that stocking rate or grazing intensity in animal populations may influence animal tissue δ^15^N values (Britton et al., 2008; Oelze et al., 2011; Makarewicz, 2014; Müldner et al., 2014). The effects of grazing on N cycling in plant communities is complex and a closer examination of the literature reveals that unlike the very consistent increase in plant δ^15^N values caused by manuring, there is not a simple relationship between grazing intensity or stocking rate and plant δ^15^N.

Studies presenting N isotopic compositions for plants and soils under different levels of grazing pressure or stocking rates are summarized in Table 2. Most studies analyzed above ground plant tissues and soil δ^15^N, and although results vary considerably across studies the general pattern observed is that more intensively grazed areas tend to have higher plant and soil δ^15^N values. This pattern of general ^15^N enrichment in more intensively grazed zones may be the product of increased N cycle openness (Ruess and McNaughton, 1987). Aside from the direct addition of mineralized or highly labile forms of N, more intensively grazed areas tend to be characterized by one or more of the following: higher rates of ammonification, leaching of NO^−^_3_, NH_3_ volatilization, and denitrification (McNaughton et al., 1988; Ruess and McNaughton, 1988; Hobbs, 1996; Frank and Zhang, 1997; Frank et al., 2004). All of these processes, with the exception of ammonification, are associated with ^15^N enrichment in the residual substrates (Robinson, 2001), and therefore by increasing both N *inputs* and *outputs* (Singer and Schoenecker, 2003), herbivores tend to cause higher δ^15^N values in soils and plants. These patterns fit with the general observation that ecosystem δ^15^N tends to be higher where inputs, cycling, and outputs are higher (Högberg and Johannisson, 1993; Högberg et al., 2011, 2014).

**Table 2:** **Summary of studies examining the effects of grazing intensity or stocking rate on plant and soil N isotopic compositions**.

**Reference**	**Type**	**Region**	**Herbivore(s)**	**Material(s) sampled^1^**	**Summary of *δ*^15^N findings**
Han et al., 2008	Meadow steppe	Inner Mongolia, China	Dairy cattle	AV, BV, S	δ^15^N_AV_ decreased with grazing intensity for non-legumes (+3 to +11‰ in lightly grazed to −3.3 to −1.3‰ in heavily grazed), but was unaffected for legumes. Inconsistent patterns for δ^15^N_BV_ and δ^15^N_S_
Sjögersten et al., 2010	Wet and mesic tundra	Arctic Norway	Barnacle goose	AV	δ^15^N_AV_ higher in grazed areas for mosses and dwarf shrubs, but not for grasses
Coetsee et al., 2011	Semi-arid savanna	East Africa	Various	AV, S	Higher δ^15^N_AV_ and δ^15^N_S_ in more intensively grazed areas (grazing lawns) relative to less intensively grazed areas (tall grass areas) but only approximately 1‰ difference and not statistically significant
Craine et al., 2009a	Savanna	South Africa	Various	AV, S	Higher δ^15^N_AV_ in protected areas with greater grazing pressure relative to areas with lower grazing pressure, but no difference for δ^15^N_S_
Wittmer et al., 2011	Semi-arid steppe	Inner Mongolia, China	Sheep	AV, S	No correlation between sheep stocking rate and δ^15^N_AV_ or δ^15^N_S_
Xu et al., 2010	Temperate grassland	Inner Mongolia, China	Sheep	AV, S	No correlation between δ^15^N_AV_ and grazing intensity. Significantly lower surface soil δ^15^N_S_ but not topsoil δ^15^N_S_ at higher grazing intensity
Aranibar et al., 2008	Various	South Africa	Cattle	AV, S	Generally higher δ^15^N_AV_ and δ^15^N_S_ in areas with higher land-use intensity (including cattle grazing) except for the most arid site
Wrage et al., 2011	Experimental pasture	Germany	Cattle	AV, S	No correlation between δ^15^N_AV_ or δ^15^N_S_ and stocking rate
Lindwall et al., 2013	Subarctic dry heath	Sweden	Reindeer	AV, BV	Forb: δ^15^N_AV_ values significantly higher in grazed vs. ungrazed areas; higher δ^15^N_BV_ but difference not significant Dwarf shrub: δ^15^N_AV_ and δ^15^N_BV_ higher in grazed areas but difference not significant
Li et al., 2009, 2012	Grassland	Alberta, Canada	Cattle	AV, L	Higher δ^15^N_AV_ and δ^15^N_L_ in moderately and heavily grazed plots relative to control plots
Cook, 2001	Semi-arid savanna	Central Australia	Cattle	AV, S	Higher δ^15^N_AV_ values in grazed sites relative to ungrazed sites for grasses; no difference in δ^15^N_AV_ (trees) or δ^15^N_S_
Mudge et al., 2013	Experimental pasture	New Zealand	Sheep	AV, S	Higher δ^15^N_AV_ and δ^15^N_S_ values in grazed sites relative to control sites. Note that grazed sites also received superphosphate and lime fertilizer treatments whereas the control site did not
Neilson et al., 1998	Upland pasture	Scotland	Sheep	AV, S	No significant difference in δ^15^N_AV_ or δ^15^N_S_ between grazed and ungrazed plots. Note that grazed plots also received chemical fertilizer applications (N:P:K 20:20:20) whereas ungrazed plots received no fertilizer
Frank and Evans, 1997	Shrub-grassland	Montana, USA	Various	AV, S	Lower δ^15^N_AV_ and higher δ^15^N_S_ values in grazed areas relative to ungrazed areas
Hawke, 2001	Agricultural pasture	New Zealand	Cattle and Sheep	V, S	No difference in δ^15^N_V_ between grazed and ungrazed sites. δ^15^N_S_ was consistently higher at grazer plots relative to ungrazed sites
Schulze et al., 1998	Open forest or *Spinifex* woodland	Northern Australia	Not stated	AV	Significantly higher δ^15^N_AV_ at heavily and moderately grazed sites relative to ungrazed sites. Note that grazing intensity was not explicitly quantified and was estimated based on grass and weed cover (see Austin and Sala, 1999)
Li et al., 2008	Desert steppe	Inner Mongolia, China	Sheep	AV, S	No correlation between δ^15^N_AV_ or δ^15^N_S_ and grazing intensity
Kriszan et al., 2014	Grassland farms	Germany and Austria	Cattle	V, S	Significantly higher δ^15^N_V_ and δ^15^N_S_ at high N input relative to low N input farms

1 Sample abbreviations as follows: AV, above-ground vegetation; BV, below-ground vegetation; V, whole plant samples or differentiation of above and belowground tissues not specified; S, soil; L, litter.

While a trend toward higher plant and soil δ^15^N with higher grazing intensity exists, several studies have demonstrated significantly *lower* δ^15^N values in plant shoots relative to roots and/or soil in areas of high NH_3_ volatilization (Frank and Evans, 1997; Erskine et al., 1998; Frank et al., 2004; Han et al., 2008), suggesting that leaves actively absorb ^15^N-depleted NH_3_ and assimilate it into organic N (Vallano and Sparks, 2013). In agricultural contexts the emission of NH_3_ and N_2_O can be very high in areas with large amounts of manure, such as occurs with intense grazing (Amon et al., 2001). The uptake of these isotopically light gaseous N compounds by plant leaves may have a mediating effect against the uptake of ^15^N-enriched soil compounds that occur with heavy grazing, and cause significant spatial variability in foliar δ^15^N values within and between fields if grazing intensity and the distribution of animal waste is uneven (see Erskine et al., 1998).

Because archaeological studies overwhelmingly analyze animal skeletal or dental tissues for their N isotopic compositions (rather than plant materials), an important consideration is whether or not the elevated δ^15^N values with higher grazing intensity/stocking rate are also reflected in animal tissues. From a strictly theoretical perspective, if the soil and plant δ^15^N values increase with increased stocking rate, so too should the δ^15^N values of herbivores grazing in these ecosystems. Wittmer et al. (2011) found no relationship between stocking rate and sheep tissue δ^15^N, but they also did not detect any significant differences in vegetation or soil δ^15^N with variable stocking rate; similar results were obtained by Wrage et al. (2011) with cattle. Schwertl et al. (2005) and Kriszan et al. (2014) found that higher grazing intensities were correlated with higher animal tissue δ^15^N values, with differences between stocking rates as high as 4‰ in both studies. This is strongly suggestive that such a pattern may also be present in the tissues of archaeological animals, although one wonders to what extent results derived from modern confinement dairy farms (with very high stocking rates) are in any way a realistic analog for ancient animal management systems. A more conservative and appropriate strategy for ancient contexts might be to compare data derived from small-scale organic farms with variable stocking rates.

### Foddering

The type of foods that animals consume is one of the many behavioral changes that may differentiate wild and domestic species. This control exerted over the diet of animals through spatial and behavioral restrictions is an important, and perhaps a defining, aspect of relations between humans and livestock. Indeed, in some areas of the world there may have been some degree of symbiotic development of animal and crop husbandry, with animal excreta being instrumental in maintaining soil fertility and cultivated plants (or byproducts) providing valuable fodder for animals (Charles et al., 1996). Many studies have interpreted isotopic data from prehistoric animals within the context of foddering strategies (Finucane et al., 2006; Madgwick et al., 2012; Makarewicz, 2014), although most have focused on variable C_3_ and C_4_ plant consumption on the basis of carbon isotopic data. There are, however, some important considerations with respect to N isotopic compositions of agricultural plants within the context of animal foddering and some processes that would be expected to significantly influence δ^15^N values in plants and animals.

The types of plants used as animal fodder are highly variable. In prehistoric Europe, when forests were much more widespread than in the present day, tree leaves were an important source of animal fodder (Regnell, 2002). While some crops may have been grown specifically to be fed to animals (Ross and Zutter, 2007), the most common source of animal fodder is agricultural byproducts, typically the stems and leaves remaining after the harvest of grains (Jones, 1996). In these instances where animals and humans consume different parts of the same plant, there are important isotopic consequences driven by N isotopic variability within plants. This is especially important because animal N isotopic data are often used for comparative or baseline purposes to assess the importance of plant- and animal-derived protein in human diets (Privat et al., 2002; Müldner and Richards, 2005).

Most of the preceding discussion has focused on factors influencing the N isotopic composition of N species taken up by plants, but there are also processes that occur within plants that have the potential to alter their N isotopic composition. Even plants grown under controlled conditions with a single N source have displayed considerable (up to 7‰) within-plant variation in δ^15^N (Yoneyama et al., 1986; Yoneyama and Kaneko, 1989; Evans et al., 1996; Robinson et al., 2000; Kolb and Evans, 2002; Szpak et al., 2012a). One reason for this within-plant variation is the assimilation of NH^+^_4_ into organic N occurs only in the roots, but the assimilation of NO^−^_3_ occurs both in the roots and the shoots. This is significant because the NO^−^_3_ that is moved to the shoot and assimilated there has already undergone some fractionation in the roots (due to the assimilation of NO^−^_3_ in the root) and is enriched in ^15^N relative to the NO^−^_3_ pool that was assimilated in the root (Evans et al., 1996). Thus, where NO^−^_3_ is the predominant N source, there exists a possibility of shoots being enriched in ^15^N relative to roots (Yoneyama and Kaneko, 1989; Evans et al., 1996; Evans, 2001).

More pertinent to the foddering of animals, however, is that when N that has been previously acquired is remobilized to areas of new growth, this may result in fractionation (Gebauer et al., 1994; Näsholm, 1994; Szpak et al., 2012a; Kalcsits and Guy, 2013). There tends to be a difference in δ^15^N between tissues that act as nitrogen sinks (e.g., grains that form during reproductive growth when vegetative growth has slowed or ceased) and nitrogen sources (e.g., leaves and stems that may reallocate much of their N to reproductive tissues such as flowers and fruits). There are two reasons that this variation occurs. First, because metabolic pathways leading to the synthesis of different amino acids are characterized by differing levels of fractionation against ^15^N (Werner and Schmidt, 2002), the selective import or export of particular amino acids between plant tissues may contribute to this intraplant variation (Tcherkez, 2011; Gauthier et al., 2013). Similarly, alterations in the distribution of proteins, free amino acids, amino sugars, and alkaloids between different tissues or organs may drive this variability because proteins tend to be ^15^N enriched relative to bulk cell N, while the other compounds listed tend to be relatively depleted in ^15^N (Werner and Schmidt, 2002). Second, the fractionations associated with the catabolism and eventual reassimilation of various N compounds (deamination and transamination) could also influence the δ^15^N values of source and sink tissues (Macko et al., 1986, 1994; Yoneyama et al., 2003) as N is remobilized during reproductive growth.

**Figure 8:**
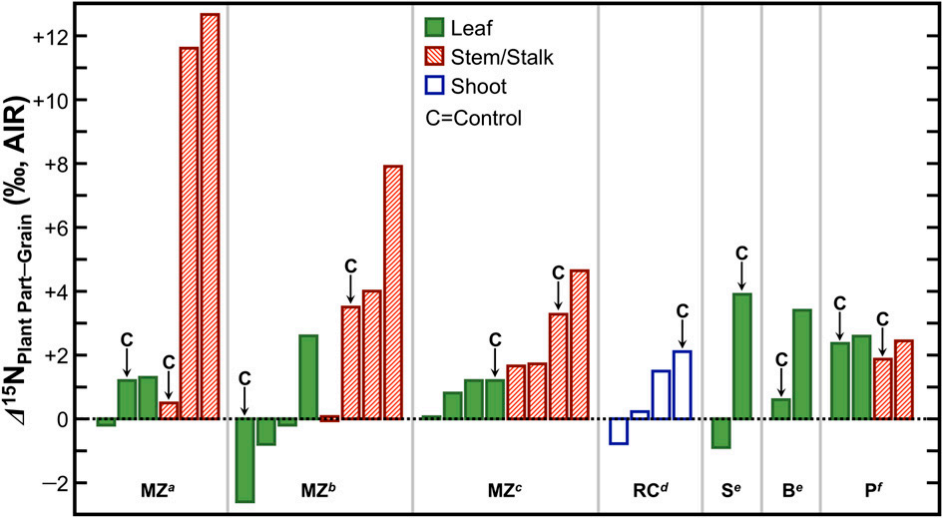
**Differences in *δ*^15^N between grains/fruits and above-ground plant parts**. The grain or fruit tends to be depleted in ^15^N relative to the leaves and stems/stalks. Data are from studies examining effects of fertilizers on plant δ^15^N values and a “C” above a particular bar denotes that these data are from a control treatment in which no fertilizer was applied. Abbreviations at the bottom of the figure denote species represented and source for data. MZ, maize; RC, rice; S, squash; B, bean; P, pepper. Data obtained from: ^*a*^Szpak et al. (2012a), ^*b*^Szpak et al. (2012b), ^*c*^Choi et al. (2002), ^*d*^Yun et al. (2011), ^*e*^Szpak et al. (2014), ^*f*^Del Amor et al. (2008).

Grains or fruits tend to act as strong N sinks. The amount of N that is remobilized to the grain from previously absorbed N is substantial, up to 85% in maize (Ta and Weiland, 1992), 100% in wheat (Martre et al., 2003; Tahir and Nakata, 2005), and 65% in rice (Mae and Ohira, 1981). Thus, fruits and grains should be systematically depleted in ^15^N relative to whole plants, leaves, and stems. In support of this notion, several studies have found lower δ^15^N values in grains relative to the leaves, stems, or shoots (Figure 8), the magnitude of which varies strongly with growing conditions, but is typically on the order of 1–4‰. Therefore, if animals were foddered to a significant extent on agricultural byproducts and humans consumed variable proportions of grains and those animals, there should be a convergence in the nitrogen isotopic compositions of the human and animal tissues relative to humans consuming variable proportions of grains and animals grazing on open pastures. This must be kept in mind when contemporaneous human and animal δ^15^N data are directly compared for paleodietary reconstructions, and considered as a possibility if higher than expected δ^15^N values are recorded in domestic animal tissues. Finally, agricultural systems tend to be characterized by higher N inputs (and in turn higher N losses) than non-agricultural systems, and are thus expected to be more prone to the loss of ^14^N (Kriszan et al., 2014). Therefore, animals foddered to a large extent on agricultural products or byproducts should be characterized by higher tissue δ^15^N values than animals grazing on wild pastures.

### Areas for future research

The potential influence of both stocking rate and foddering require much more research to resolve what influence they may actually have on animal tissue N isotopic compositions. As suggested previously for agricultural practices, considerable potential lies in the isotopic analysis of animal tissues derived from small-scale or traditional herders. In many regions, particularly in Europe, where the production of particular kinds of meats, cheeses, and other animal products is regulated and occurs on relatively small or at least non-industrial scales, there is potential to investigate a wide-variety of animal management strategies that have been maintained over long periods of time. Certainly isotopic analysis have already been used extensively in attempts to verify the geographic origin of particular animal products (reviewed by Gonzalvez et al., 2009), but more directed efforts should also be made to assess the consequences of different management techniques on animal tissue (and potentially plant tissue) isotopic compositions (Von Holstein et al., 2013).

## Summary and concluding remarks

A large number of environmental and cultural variables may strongly influence the N isotopic compositions of plant-soil systems. With respect to agricultural systems, the following generalizations can be made about the three areas focused on in this paper:

1. *Animal Fertilizers.* The use of animal fertilizers will increase plant δ^15^N values by a variable amount depending on the type of fertilizer applied, the amount applied, and the duration of application. For some fertilizers (such as cattle manure) the effect is relatively small, on the order of +2 to +8‰, while the effect is substantial for others such as pig manure (+15 to +20‰) or seabird guano (+10 to +40‰).
2. *Burning/Shifting Cultivation.* Soil and vegetation δ^15^N values will increase in the years immediately after burning, and subsequently return to pre-fire levels. The magnitude of the difference in plant δ^15^N between recently burned and unburned vegetation may be between 2 and 8‰, although there is considerable variation.
3. *Tillage*. Ploughing or tillage is unlikely to influence plant δ^15^N in agricultural fields because these soils are typically not characterized by the large depth-related variation in δ^15^N values observed in forests.

The impact of animal fertilizers on plant N isotopic compositions has been relatively well investigated insomuch as it is now firmly established that plant N isotopic compositions consistently increase with manuring. While additional studies in this area would certainly be useful, more attention must be paid to other aspects of prehistoric cultivation practices (e.g., tillage, crop rotations, irrigation and floodplain agriculture, intercropping), and how they might affect soil and plant δ^15^N values. The end product will likely be a more complicated pattern of N isotopic variation with relatively few consistent and predictable effects for individual processes. At the very least, this will result in the ability to better and more accurately convey these complexities and incorporate various levels of uncertainty into reconstructions of ancient diet and agricultural practices.

With respect to animal husbandry, the following generalizations can be made about the two areas focused on in this paper:

1. *Grazing Intensity/Stocking Rate.* Generally, with increased grazing intensity, plant and soil δ^15^N values tend to increase. Some experimental work with modern animals has also shown that their tissues are positively correlated with stocking rate, although it is not clear whether or not these rates are reasonable proxies for ancient animal management regimes.
2. *Foddering*. The foddering of domestic animals with agricultural byproducts may increase animal tissue δ^15^N values because plant parts (leaves and stems) that supply N to reproductive structures are typically enriched in ^15^N relative to grains. Currently there is no supporting evidence for this notion from studies of modern animals foddered on agricultural byproducts.

Additional research focusing on the isotopic consequences of different animal management strategies in modern contexts would be useful for the interpretation of isotopic data derived from ancient animal populations.

Given the extreme complexities of N isotopic biogeochemistry in plant-soil systems and the multitude of factors that may influence plant δ^15^N values, how do we move forward in dealing with N isotopic data from ancient contexts? While early efforts in the field established general patterns that were useful for the qualitative interpretation of isotopic data (DeNiro and Epstein, 1981; Schoeninger et al., 1983; Schoeninger and DeNiro, 1984), much of the work discussed in this paper has focused on how the complexities of the N cycle lead to large variation and uncertainty in plant N isotopic compositions. First and foremost, the complexities of these systems must be acknowledged and effectively communicated in archaeological literature. A simplistic treatment of N isotopic data wherein there is a trophic level effect and a distinction between marine and terrestrial environments inadequately captures the nature of this variation. With respect to data treatment, recent mixing models that utilize a Bayesian framework have the capacity to incorporate uncertainty in source parameters (Moore and Semmens, 2008; Parnell et al., 2010) and because of this, these models have the potential to more realistically convey these baseline complexities and uncertainties, although quantitative mixing models have not been embraced by archaeologists to date (but see Kellner and Schoeninger, 2007). These models certainly have limitations and any dietary reconstruction of ancient populations is necessarily fraught with considerable uncertainty, but there is greater potential to more honestly communicate this uncertainty to the non-specialist via the Bayesian approaches. Methodologically, additional work focused on the N isotopic analysis of individual amino acids isolated from bone collagen (Naito et al., 2010) or plant remains (Styring et al., 2014) has considerable potential with respect to elucidating the relative importance of different biogeochemical processes in determining N isotopic compositions, but this field is still in its infancy.

A diverse array of factors can influence plant and animal δ^15^N values. Because of this, we need to explicitly consider N isotopic measurements as integrators of the complexities of the N cycle (Robinson, 2001) rather than tracers of individual processes. With respect to archaeological samples, in the absence of abundant supporting evidence, it is rare that one process can be singled out as causative for any pattern. Generally speaking, N isotopic compositions are rarely well positioned to directly answer *specific* questions about many processes that may be of interest to archaeologists, but are most effective when integrated with other lines of evidence.

### Conflict of interest statement

The author declares that the research was conducted in the absence of any commercial or financial relationships that could be construed as a potential conflict of interest.
